# Mitochondrial DNA content and mass increase in progression from normal to hyperplastic to cancer endometrium

**DOI:** 10.1186/1756-0500-5-279

**Published:** 2012-06-07

**Authors:** Antonella Cormio, Flora Guerra, Gennaro Cormio, Vito Pesce, Flavio Fracasso, Vera Loizzi, Leonardo Resta, Giuseppe Putignano, Palmiro Cantatore, Luigi Eustacchio Selvaggi, Maria Nicola Gadaleta

**Affiliations:** 1 Department of Biosciences, Biotechnologies and Pharmacological Sciences, University of Bari, Via Orabona, Bari, 4-70126, Italy; 2 Department of Gynecology, Obstetrics and Pediatrics, Unit of Medical Genetics, University Hospital S.Orsola-Malpighi, Via Massarenti 9, Bologna, 40138, Italy; 3 Department of Gynecology, Obstetrics and Neonatology, University of Bari, Piazza Giulio Cesare, Bari, 11-70124, Italy; 4 Department of Pathological Anatomy, University of Bari, Piazza Giulio Cesare, Bari, 11-70124, Italy; 5 National Research Council of Italy, Institute of Biomembranes and Bioenergetics, Via Amendola, Bari, 165/A-70126, Italy; 6 Centro di Eccellenza di Genomica in Campo Biomedico ed Agrario, University of Bari, Bari, Italy

**Keywords:** Endometrial cancer, Typical hyperplasia, Atypical hyperplasia, MtDNA content, Citrate synthase activity

## Abstract

**Background:**

An increase in mitochondrial DNA (mtDNA) content and mitochondrial biogenesis associated with the activation of PGC-1α signalling pathway was previously reported in type I endometrial cancer. The aim of this study has been to evaluate if mtDNA content and the citrate synthase (CS) activity, an enzyme marker of mitochondrial mass, increase in progression from control endometrium to hyperplasia to type I endometrial carcinoma.

**Results:**

Given that no statistically significant change in mtDNA content and CS activity in endometrium taken from different phases of the menstrual cycle or in menopause was found, these samples were used as control. Our research shows, for the first time, that mtDNA content and citrate synthase activity increase in hyperplastic endometrium compared to control tissues, even if their levels remain lower compared to cancer tissue. In particular, mtDNA content increases seem to precede increases in CS activity. No statistically significant change in mtDNA content and in CS activity was found in relation to different histopathological conditions such as grade, myometrial invasion and stage.

**Conclusion:**

MtDNA content and citrate synthase activity increases in pre-malignant lesions could be a potential molecular marker for progression from hyperplasia to carcinoma.

## Background

The most common malignancy in the female genital tract, endometrial carcinoma is classified in two main types: estrogen-related (type I, endometrioid) and non–estrogen-related (type II, nonendometrioid) [1]. Type II tumors behave aggressively and lack the progesterone responsiveness of type I tumors. The development of endometrioid cancer is usually considered a multistep process with slow progression from normal endometrium to hyperplasia to cancer as a result of excesses in endogenous or exogenous estrogens and**/**or relative progesterone deficiency [2].

Endometrial hyperplasia is a pathologic condition characterized by proliferation of endometrial glands with a greater than normal gland-to-stroma ratio. About 1.6% of patients diagnosed with these abnormalities can develop endometrial cancer. Endometrial hyperplasia may also be associated with cytologic atypia (atypical hyperplasia). While these features are similar to those seen in true cancer cells, atypical hyperplasia does not show invasion into the connective tissues, the defining characteristic of cancer [3]. About 22% of patients with atypical hyperplasia can develop cancer [4].

Mitochondria, which are essential organelles that generate cellular energy (ATP) through oxidative phosphorylation, have long been suspected to play an important role in the development and progression of cancer. Warburg has hypothesized that a key event in carcinogenesis is the development of an ‘injury’ to the respiratory machinery that results in compensatory increases in glycolytic ATP production [5]. The respiratory machinery is the product of two genomes, nuclear genome and mitochondrial genome. Mitochondrial DNA (mtDNA) is a circular double-stranded molecule of 16569 bp that codes for two rRNAs, 22 tRNAs and only 13 of about 100 proteins in the mitochondrial respiratory chain complexes [6].

Recently, changes in mtDNA content have been investigated in solid tumors. A depletion of mtDNA induces cancer progression in prostate, ovarian and breast cancer [7-9]. Several studies have revealed an increased mtDNA content in prostate [10], endometrial [11], and pancreatic cancer [12] as well as in thyroid and renal oncocytoma [13,14].

An increase in mtDNA content and citrate synthase (CS) activity [15], an enzyme marker of mitochondrial mass [16], has been reported in type I endometrial cancer compared to proliferative endometrial control tissues, suggesting an increase in mitochondrial biogenesis. A statistically significant increase in TFAM, NRF-1 and PGC-1α protein content, key factors in the PGC-1α dependent mitochondrial biogenesis signalling pathway, was found, moreover, in endometrial cancer tissue compared to controls. This suggests that the increased mitochondrial biogenesis in type I endometrial cancer can be associated with upregulation of PGC-1α signalling pathway [15]. MtDNA mutations, associated with increases in mitochondrial biogenesis, which result in the disassembly of respiratory chain complexes, were noted in type I endometrial cancer tissues for the first time. MtDNA mutations did not occur in typical hyperplastic or atrophic tissue surrounding tumour in the same patient [17].

Since hyperplasia often precedes type I endometrial carcinoma, it is vital to establish if increases in mtDNA and/or CS activity appear early in hyperplastic endometrium and if these changes can be considered possible markers for progression from benign to premalignant lesions. For this reason mtDNA content and CS activity were measured in endometrial tissue taken from different phases of the menstrual cycle (proliferative, secretory) or in menopause (atrophic), in hyperplastic (atypical or typical) and cancer tissue.

## Results

### Analysis of mtDNA content in control, hyperplastic and cancer endometrial tissues

MtDNA content relative to nuclear DNA content (mtDNA/nDNA) in 16 proliferative, 7 secretive and 9 atrophic endometria was measured by real-time PCR. No statistically significant difference was found in the mean value of mtDNA/nDNA ratio between proliferative (mean ± SD = 286 ± 96), secretive (mean ± SD = 316 ± 98) and atrophic endometria (mean ± SD = 323 ± 206). These three types of endometrium were therefore considered all together and the mean value of their mtDNA/nDNA ratios was used as the control value.

**Figure 1:**
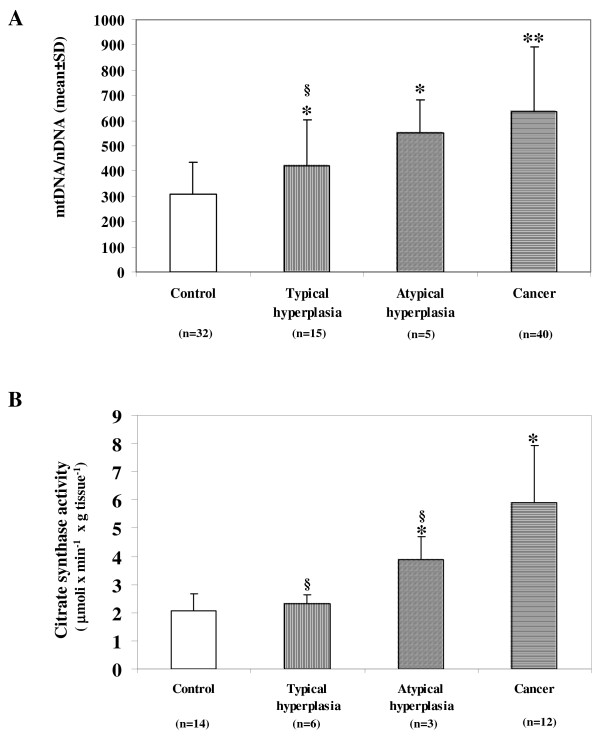
**A) MtDNA content in control, typical hyperplasia, atypical hyperplasia and cancer.** MtDNA content has been measured as mtDNA/nDNA ratio. Bars represent the average ± standard deviation of values obtained from different samples analysed in triplicate in 3–4 experiments. n, number of analysed samples. B) Citrate synthase activity in control, typical hyperplasia, atypical hyperplasia and cancer. Measurements of citrate synthase activity (μmoli x min^-1^x g tissue^-1^) were performed in cellular homogenate. Bars represent the average ± standard deviation of values obtained from different samples analysed in triplicate in 3 different experiments. *p < 0.05 and **p < 0.001 statistically significant differences versus control. § p < 0.05 statistically significant difference versus cancer.

The mtDNA/nDNA ratio was measured in 20 typical and atypical hyperplastic tissues and in 40 samples of type I endometrial adenocarcinoma. Figure 1A shows a statistically significant increase in 1.4, 1.8 and 2-fold of mtDNA content respectively in typical hyperplasia, atypical hyperplasia and cancer compared to control. Compared to cancer tissue mtDNA content is lower in typical and atypical hyperplastic samples, but only statistically significant for typical hyperplasia.

### Citrate synthase activity in control, hyperplastic and cancer endometrial tissues

To verify if increases in mtDNA content are associated with increases in mitochondrial mass, CS activity was measured in the homogenate of 16 endometrial control samples (8 proliferative and 6 atrophic endometria), 9 hyperplastic samples that were divided into typical and atypical hyperplasia, and 12 endometrial cancer samples. No statistically significant difference was found in the mean value of CS activity between proliferative (mean ± SD = 2.16 ± 0.79) and atrophic endometria (mean ± SD =1.96 ± 0.42). These two types of endometrium were therefore considered all together and the mean value of their CS activity was used as the control value. Figure 1B shows that there is a statistically significant 2 and 3- fold increase in CS activity compared to the control tissues in atypical hyperplasia and in cancer tissue respectively. In typical and atypical hyperplasia, however, CS activity is significantly lower compared to cancer tissue samples.

### MtDNA content and citrate synthase activity in cancer progression

MtDNA content and CS activity in cancer tissue samples were analyzed in relation to different histopathological conditions such as grade, myometrial invasion and stage. No statistically significant change in mtDNA content and CS activity was found in cancer progression (data not shown). At the more advanced stage (stage IV), and with highest grade (G3) tumours, however, mtDNA content seemed to decrease compared to stage 1 and grade 1 tumors (respectively for stage 486±56 vs 645±31 and for grade 503± 71 vs 689±67). No evaluation of CS activity was possible in relation to cancer stage due to limited availability of tissue samples.

## Discussion

Diagnosis of premalignant lesions in routine endometrial biopsies has great clinical value in patient management. A number of possible molecular markers (PTEN, Bcl-2, Bax) have been analysed to establish the risk of malignant transformation in endometrial hyperplasia [18,19]. Since there is no reported data concerning mtDNA content and CS activity in hyperplastic tissue and endometrial carcinogenesis, we have tried to establish if changes in mtDNA content and/or CS activity could be potential molecular markers of progression from hyperplasia to carcinoma. To this end mtDNA content and CS activity were measured in normal endometrium from different phases of the menstrual cycle, in hyperplasia and cancer. MtDNA was expressed as a mtDNA/nuclearDNA ratio. MtDNA, in fact, replicates independently from nDNA so that copies of mtDNA per cell can change under different cell conditions [6]. Since changes in the ploidy have rarely been reported in hyperplasia and type I endometrial cancer [20], the mtDNA/nDNA ratio could indicate increases or decreases in mtDNA copy number per single cell.

Our research shows that mtDNA content and CS activity increase in progression from benign endometrium to hyperplasia and to carcinoma. MtDNA content increases precede increases in CS activity. While mtDNA could therefore be considered as one of the factors that begin to increase early in typical hyperplasia, it is more evident in atypical hyperplasia where malignant transformation is more probable. CS activity was found to increase only in atypical hyperplasia compared to control tissues. This increase could be the result of mitochondrial proliferation that probably begins with an increase in mtDNA content in typical hyperplasia.

In recent years there has been increasing evidence pointing to the mitochondrial respiratory chain (MRC) as a novel and important target for the action of estrogen and estrogen receptors (ER). ER is also localised in mitochondria while E2 up-regulates transcript levels of several mtDNA and nuclear genes encoding MRC proteins [21]. Estradiol has been reported to increase mtDNA content in breast and lung adenocarcinoma cells that directly stimulate NRF-1 gene expression and increase TFAM expression, mitochondrial transcription and oxygen consumption [22]. In human breast cancer cells, on the other hand, estradiol produces high rates of mitochondrial reactive oxygen species (ROS) that act as signal transducing factors activating NRF-1 [23]. Interestingly, enhanced lipid peroxidation and altered antioxidant enzyme activities were found in patients with endometrial hyperplasia and cancer compared to patients with benign disease [17,24]. Moderate increases in mitochondrial biogenesis reported here in endometrial hyperplasia could be either as a direct result of estrogen or a cellular attempt to counteract estrogen ROS increase. In our previous work we have found pathogenetic mtDNA mutations in type I endometrial cancer tissue associated with more evident increases in mitochondrial biogenesis compared to typical hyperplastic or atrophic tissue surrounding tumours and the activation of oxidative stress response mechanisms [17]. In cancer tissue induction of mitochondrial biogenesis might also be explained as a compensatory response to pathogenic mtDNA mutations.

No statistically significant differences in mtDNA content and CS activity were found in tumour samples classified according to known prognostic factors (grade, depth of myometrial invasion and stage). A slight decrease in mtDNA content, while not statistically significant, seemed, however, to be present in high grade tumours. This trend can be explained by the fact that high grade tumours are less exposed to estrogen and are characterised by a high rate of cell division which could in turn be responsible for the lower number of mitochondria per cell.

## Conclusions

We note here for the first time moderate increases in mitochondrial biogenesis in hyperplastic tissues probably linked to estrogen stimulation. Of the two types of hyperplasia atypical hyperplasia seems to be more affected by this stimulation than typical hyperplasia. A more significant increase in mitochondrial biogenesis is found in cancer tissue probably as a compensatory response to damage caused by mtDNA mutations. MtDNA mutations would seem therefore to enhance estrogen stimulation as a synergic mechanism.

Different levels in mtDNA and CS activity, therefore, could be used as possible molecular markers to establish the risk of malignant transformation in endometrial hyperplasia.

## Methods

This study was carried out using endometrial tissue samples obtained from patients who underwent laparotomic hysterectomy between January 2007 and June 2008 at the Department of Gynecology, Obstetrics and Neonatology, University of Bari, Italy. Following surgery, tissues were snap-frozen in liquid nitrogen and stored at −80°C. None of the patients had received any treatment (radiotherapy, chemotherapy or hormone therapy) before surgery. Endometrial tissue samples were analysed from patients in different phases of the menstrual cycle (16 proliferative and 7 secretive) or in menopause (9 atrophic) who underwent hysterectomy for benign conditions such as uterine prolapse and ovarian cysts. The mean age of these patients is 51 ± 9 years (range 37 to 78). Endometrial hyperplasia samples (5 atypical, 15 typical) were taken from 6 patients who underwent hysterectomy for hyperplasia diagnosis and from 14 patients affected by hyperplasia and type I endometrial carcinoma. The mean age of these patients is 65 ± 13 years (range 46 to 86). All endometrial samples were characterised histologically. Neoplastic endometrial tissue samples were analysed from 40 patients affected by type I endometrial carcinoma. The mean age of these patients is 69 ± 12 years (range 46 to 86). Stage, miometrial invasion and grading of endometrial carcinoma were assigned according to categories devised by the International Federation of Gynecology and Obstetrics (FIGO), 2007 and 2008 [25]**.** All clinical samples were collected under protocols approved by Institutional Review Board of the University Hospital of Bari, Italy.

Total DNA was prepared from endometrial tissue using NucleoSpin Tissue Kit (Machinary-Nagel) and stored at −20°C. MtDNA content was measured by real-time PCR using an ABI Prism 7000 real-time PCR (Applied Biosystems). As previously reported [15], mtDNA content was related to nuclear DNA (nDNA) amount. The difference in threshold cycle values of nuclear Beta Actin gene and mitochondrial ND1 gene (ΔCt, namely Ct_ND1_–Ct_Actin_) was used as a measure for the relative abundance of the mitochondrial genome. The mtDNA/nDNA ratio is reported as 2^-ΔCt^.

Total proteins were extracted from 20 mg of frozen endometrial samples. Citrate synthase activity (μmol x min^–1^ x g tissue^–1^) was measured according to [15]

Statistical analysis was carried out using SPSS software. ANOVA test or t-student’s test was used. Statistical significance was set at *p <* 0.05.

## Abbreviations

(mtDNA): Mitochondrial DNA; (CS): Citrate synthase.

## Authors’ contributions

Research project was devised by AC. AC was also involved in writing and revising the article and measuring mtDNA content. FG was involved in measuring mtDNA content. GC, VL and GP carried out analysis of patients who underwent surgery at the Department of Gynecology, Obstetrics and Neonatology, University of Bari. VP and FF were involved in measuring CS activity. LR carried out histological analysis. MNG, PC and LS were involved in writing and editing article and interpreting data. All authors read and approved the final manuscript.

## Competing interests

The authors have no competing interests.
